# Primary extraskeletal osteosarcoma in a bladder diverticulum

**DOI:** 10.1016/j.eucr.2022.102214

**Published:** 2022-09-07

**Authors:** Reilly Carr, Michael Hsueh-Ching Hsia, Joseph Grossman

**Affiliations:** a Tripler Army Medical Center, Honolulu, HI 96859, USA; b Department of Urology, Medical University of South Carolina, Florence, SC 29505, USA; c Pee Dee Pathology Associates, 805 Pamplico Hwy, Suite B-210, Florence, SC 29505, USA

**Keywords:** Extraskeletal, Osteosarcoma, Bladder diverticulum, Soft-tissue tumor, Cystoscopy

## Abstract

Extraskeletal osteosarcoma (ESOS) occurs when an osteosarcoma presents in a primary location outside of the bone. These account for only 1% of all sarcomas. We present the case of a 78-year-old male with palpable right lower quadrant mass who had ESOS in a bladder diverticulum. Less than 50 cases of ESOS in the bladder have been reported. This marks the fourth case of primary osteosarcoma found within a bladder diverticulum.

## 1 Introduction

Osteosarcoma is a mesenchymal tumor characterized by the presence of malignant osteoblasts which erroneously produce osteoid. Normally, OS occurs at the metaphyseal growth plate of long bones such as the femur. The distribution of ages affected by OS is traditionally bimodal, with peak occurrences in both adolescence and adulthood after the age of 50.^1^ Very rarely, OS can occur in other soft tissues without a primary skeletal site. This is known as extraskeletal osteosarcoma. ESOS accounts for only 1% of soft tissue sarcomas. ESOS to the bladder is exceedingly rare, with less than 50 cases reported in the literature. We present the case of ESOS within a palpable bladder diverticulum in a 78-year-old male, which was found during cystoscopy. This case is the fourth reported ESOS within a bladder diverticulum.

## 2 Case description

A 78-year-old male was found down in his home and was brought to the emergency department (ED). He exhibited confusion and mental status changes. Past medical history included recent NSTEMI (Non-ST elevation myocardial infarction) and CVA (cerebrovascular accident), but before these episodes the patient had not seen a medical provider for several decades. He had no previous surgeries. He never smoked but did consume alcohol heavily in the past. He spent most of his life working in a textile factory, though it is unclear what occupational exposures, if any, he may have had at this site. The patient had a strong family history of prostate cancer and breast cancer on his mother's side. On physical exam the patient was noted to have suprapubic abdominal distention and a palpable, hard, mass in the right lower quadrant.

**Fig. 1:**
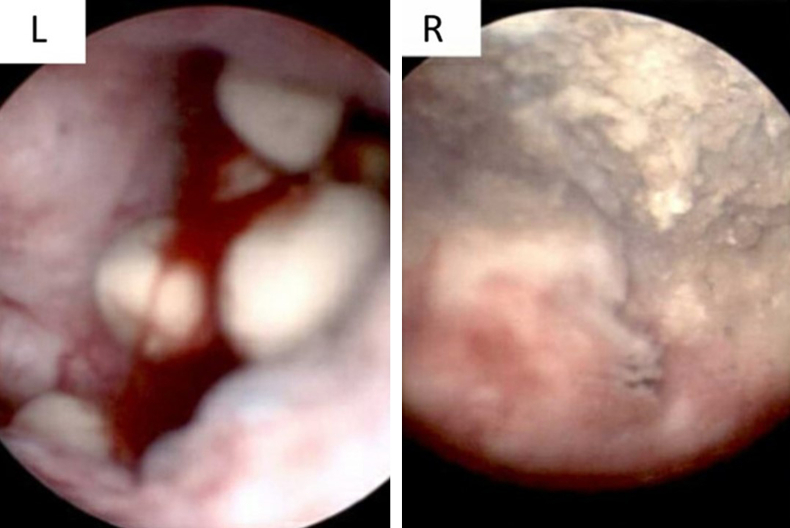
Cystoscopic images of diverticulum. Courtesy of Dr. Michael Hsia^2^ Left: View of the diverticulum from outside. Some calculi that have accumulated can be seen. Right: View from within the diverticulum. The calcified shell of the diverticulum is clearly visible, as well as one of the masses which was biopsied at the periphery of the calcification.

ED staff were unable to place a foley catheter for bladder decompression. Urology was consulted. The patient was taken to the operating room for a catheter placement under cystoscopic guidance, plus examination of urethra and bladder. Severe BPH was noted during navigation through the urethra. Once in the bladder, multiple trabeculations were noted, as well as a right sided bladder diverticulum with a calcified shell. A few calculi were noted around the entrance to the diverticulum (Figure 1L). Once interrogated further, a pulsatile mass and several other masses were noted in and along this calcified shell(Fig. 1R).^2^ Three biopsies were taken from masses in and around the diverticulum and sent for evaluation.

**Fig. 2:**
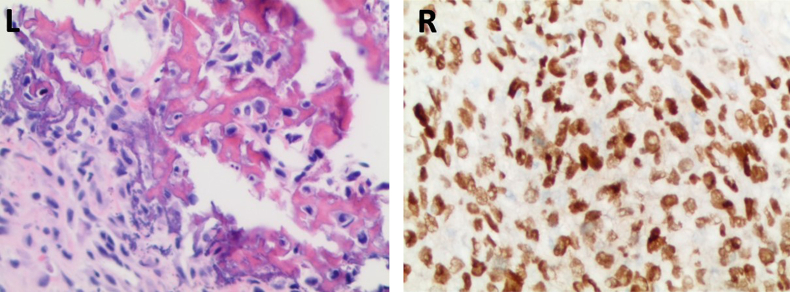
Microscopic Slides of Biopsied Tissue. Courtesy of Dr. Joseph Grossman^3^ Left: H&E stained sections demonstrate a sarcomatoid solid tumor comprised of round to oval cells with hyperchromatic nuclei of variable size, numerous scattered mitotic figures including atypical forms, and multiple foci with osteoid and calcifications. Presence of osteoid is a diagnostic requirement for osteosarcoma. Right: Immunostain for SATB2 diffusely marks lesional cell nuclei; a finding associated with osteosarcoma. Additional immunostains for pan-cytokeratins, GATA-3, SOX-10, and NKX-3.1 were all negative, tending to exclude potential mimics including de-differentiated urothelial and prostatic carcinomas, melanoma, and others.

A catheter was successfully placed intraoperatively, and the patient was admitted to the hospital. Unfortunately, the patient continued to have altered mental status throughout the hospital admission, preventing further assessment because of confusion and combativeness. Pathology report for the biopsied tissue revealed evidence of osteosarcoma (Fig. 2).^3^ A primary source of skeletal osteosarcoma was excluded with imaging and clinical exam. Potential outcomes and complications of radical cystectomy, chemotherapy, and radiation were all discussed with the patient's family. Because the patient was not improving neurologically and ESOS has a very poor prognosis, the family opted for palliative care. The patient expired two weeks later, 17 days after initial presentation to the ED.

## 3 Discussion

Bladder diverticuli are outpouchings of bladder mucosa through the muscularis layer of the bladder. They are caused by increased intravesicular pressure, commonly secondary to neurogenic bladder or bladder outlet obstruction such as a urethral stricture or BPH. The prevalence of intradiverticular tumors is somewhere between 1 and 10%. The mechanism for this relationship is postulated to be due to urinary stasis within the diverticulum, which causes mucosal irritation, as well as prolonging tissue exposure to any carcinogens in the urine.

Currently, treatment of ESOS is not well established. A retrospective study of 25 ESOS patients by Heng et al. (2020) sought to compare chemotherapy and radiation therapy for ESOS patients. They compared the outcomes of “osteosarcoma type” chemotherapy regimens, “soft-tissue sarcoma type” chemotherapy regimens, and radiotherapy regimens. They found no difference in survival or systemic recurrence between the two chemotherapy groups, and neither chemotherapy nor radiation therapy demonstrated significant disease-free or overall survival.^4^ This study excluded retroperitoneal and intraabdominal ESOS. Intravesicular ESOS is currently treated similarly to urothelial carcinoma, with cisplatin-based chemotherapy followed by radical cystectomy in many cases. A literature review by Ghalayini et al. (2001) found similarly poor prognosis for intravesicular ESOS patients as well, with 22 of 25 patients dead at 6 month follow up. Most of these deaths were due to local spread with urinary tract obstruction and secondary infection.^5^

## 4 Conclusion

ESOS of the bladder is an exceedingly rare cancer with very poor prognosis. Current treatments of ESOS of any location have not demonstrated effectiveness using established surgical procedures, chemotherapy, or radiation therapy. It has been postulated that although ESOS is a type of osteosarcoma, it may behave differently than bony OS or other soft-tissue sarcomas. Further research is needed to determine optimal treatment plans for patients diagnosed with ESOS.

## Consent

Consent provided by patient's brother as patient was deceased.

## Declaration of competing interest

No disclosures.

## Abbreviations

OS: Osteosarcoma
ESOS: Extraskeletal osteosarcoma
ED: Emergency department
